# The Impact of Prenatal Care on the Prevention of Neonatal Outcomes: A Systematic Review and Meta-Analysis of Global Health Interventions

**DOI:** 10.3390/healthcare13091076

**Published:** 2025-05-06

**Authors:** Mohammed Nasser Albarqi

**Affiliations:** Family and Community Medicine Department, College of Medicine, King Faisal University, Hofuf 36291, Saudi Arabia; aalbarqi@kfu.edu.sa

**Keywords:** prenatal care, neonatal mortality, nutrition, mental health, telehealth, low birth weight, systematic review

## Abstract

Background/Objectives: Neonatal outcomes, including low birth weight, preterm birth, and neonatal mortality, pose significant global health challenges, particularly in low- and middle-income countries. Prenatal care has emerged as a critical intervention in mitigating these risks through medical, nutritional, and psychosocial support. This study aimed to systematically assess the effectiveness of prenatal care interventions in preventing neonatal outcomes across diverse settings. Methods: A systematic review and meta-analysis were conducted according to PRISMA guidelines, with the protocol registered in PROSPERO (CRD42024601066). Fourteen peer-reviewed studies were included following a comprehensive search across five major databases. Eligible studies reported quantitative neonatal outcomes associated with prenatal care interventions, including nutritional supplementation, mental health services, telehealth, and routine antenatal care. Random-effects models were used for meta-analysis, and the risk of bias was assessed using RoB 2 and the Newcastle–Ottawa Scale. Results: Nutritional interventions, especially folic acid and iron supplementation, significantly reduced neonatal mortality by up to 40% (RR = 0.60, 95% CI: 0.54–0.68). High-quality prenatal care was associated with a 41% reduction in neonatal mortality. Psychosocial support reduced the risk of low birth weight and preterm birth, while telehealth interventions lowered NICU admissions in low-risk populations (RR = 0.88, 95% CI: 0.75–1.03). Heterogeneity was substantial (I^2^ = 70%), and publication bias was suggested. Conclusions: Comprehensive prenatal care, integrating medical, nutritional, and mental health interventions, significantly improves neonatal outcomes. The global implementation of accessible, high-quality prenatal services is essential, particularly in underserved populations, to reduce neonatal morbidity and mortality.

## 1. Introduction

In recent decades, prenatal care has increasingly been recognized as a critical public health intervention aimed at improving maternal and neonatal outcomes [1]. According to recent global statistics from the World Health Organization (WHO), approximately 2.3 million neonatal deaths occurred in 2021, with neonatal mortality remaining disproportionately higher in low- and middle-income countries (LMICs) compared to high-income regions [2]. Neonatal outcomes, including preterm birth complications, low birth weight (LBW), and congenital anomalies, significantly contribute to these mortality rates and long-term morbidity, especially in resource-limited settings [3]. Such alarming statistics underscore the pressing need for effective preventive interventions and highlight prenatal care as a pivotal strategy for reducing adverse neonatal outcomes [4].

Prenatal care encompasses a wide spectrum of interventions designed to enhance the health of pregnant women and their newborns. It typically includes medical check-ups, nutritional counseling and supplementation, psychosocial and mental health support, infection screening, and preventive measures such as immunizations [5]. Each of these interventions operates through specific physiological mechanisms to improve neonatal outcomes. For example, iron supplementation mitigates maternal anemia, thereby optimizing maternal and fetal oxygen delivery, enhancing fetal growth, and reducing risks of preterm birth and LBW [6,7]. Similarly, folic acid supplementation is crucial for preventing neural tube defects by supporting healthy cell growth and tissue formation during critical developmental periods [8]. Psychosocial interventions help manage maternal stress and anxiety, which physiologically reduces cortisol exposure, thereby decreasing the risks of preterm birth and developmental impairments [9]. Despite clear biological rationales, variability in prenatal care effectiveness across different contexts has been reported in the literature, highlighting discrepancies influenced by factors such as healthcare access, intervention adherence, socioeconomic conditions, and healthcare infrastructure quality [10].

Preterm birth complications, such as low birth weight (LBW) and congenital anomalies, not only contribute to neonatal mortality but also result in long-term morbidity. Notably, there is an increasing recognition of neonates surviving with complex medical needs, forming a growing subpopulation termed “children with medical complexity” [11]. This shift has substantial implications for pediatric care systems and further highlights the importance of effective prenatal interventions.

A significant body of research, including multiple systematic reviews and meta-analyses, has affirmed prenatal care’s effectiveness in enhancing maternal and neonatal health [12]. For instance, meta-analytic studies by Bhutta et al. (2013) and Lassi et al. (2021) demonstrated that comprehensive nutritional interventions during pregnancy significantly decrease neonatal mortality and morbidity in LMICs [13,14]. Yet, findings from other studies have sometimes challenged these conclusions, suggesting limited or variable impacts of prenatal care interventions in certain contexts or populations, which are often attributed to barriers such as inadequate healthcare infrastructure, limited access to quality care, poor adherence, or differing socioeconomic circumstances [15]. This conflicting evidence necessitates a more nuanced exploration of prenatal care interventions and their effectiveness across diverse global settings.

## 2. Materials and Methods

### 2.1. Search Strategy and Selection Criteria

This systematic review and meta-analysis adhered to the Preferred Reporting Items for Systematic Reviews and Meta-Analyses (PRISMA) guidelines to ensure a transparent and rigorous process. Following the PRISMA-P protocols, the research protocol was registered with PROSPERO (registration number: CRD42024601066), which underscores our commitment to maintaining high standards in our systematic review methodology.

A comprehensive search of electronic databases was conducted to identify studies relevant to the role of prenatal care in preventing neonatal outcomes. The databases searched included PubMed, Embase, Cochrane Library, Web of Science, and Scopus, covering studies published up to 30 April 2024. The search strategy incorporated both Medical Subject Headings (MeSHs) and relevant keywords, carefully chosen to ensure broad coverage of the literature. Key search terms included “prenatal care”, “Neonatal outcomes”, “birth outcomes”, “low birth weight”, “preterm birth”, “congenital anomalies”, “neonatal mortality”, and “public health interventions” (Table 1).

The search strategy underwent multiple iterations and refinements to ensure comprehensiveness. Boolean operators (AND; OR) were employed to combine key terms and capture all relevant studies. In addition to database searches, the reference lists of all included studies and review articles were manually searched to identify any additional eligible studies. Grey literature, such as conference abstracts, reports, and theses, was also considered to minimize the risk of publication bias.

This search was augmented by a manual search for the references section in relevant articles and grey literature. Efforts were made to retrieve unpublished studies or those still in progress and to minimize language bias by including studies published in languages other than English where translations were available.

### 2.2. Eligibility Screening

After removing duplicates, the eligibility screening process was carried out in two stages. First, titles and abstracts were independently reviewed by two authors to assess their relevance. In cases of disagreement, a third reviewer was consulted to resolve discrepancies. Studies that passed this initial screening underwent full-text review based on pre-established inclusion and exclusion criteria.

#### 2.2.1. Inclusion Criteria

- Studies involving human subjects with available data on neonatal outcomes (e.g., preterm birth, low birth weight, and congenital anomalies).
- Studies evaluating the impact of prenatal care interventions on neonatal outcomes.
- Randomized controlled trials, cohort studies, case–control studies, and systematic reviews.
- Studies published in peer-reviewed journals, including full-text papers with available data.
- Studies had to clearly report methods addressing selection and measurement biases. Research rated as “high-risk” studies in two or more domains using RoB 2 tools were excluded to maintain reliability.
- Only studies published in English were included due to practical constraints related to translation accuracy, resource limitations, and comparability in methods reporting. While this exclusion criterion potentially introduces language bias, we mitigated this risk by performing a manual search of the references from included English studies and grey literature to identify important non-English studies translated or summarized into English when available.

#### 2.2.2. Exclusion Criteria

- Non-research articles such as case reports, editorials, and conference abstracts.
- Studies focusing on animals or in vitro research.
- Studies not focused on prenatal care interventions or lacking data on neonatal outcomes.
- Non-English studies where translations were not accessible.
- Studies that did not clearly report neonatal outcomes quantitatively.
- Sample sizes that were below the specified minimum threshold (<100 participants).
- A high risk of bias identified in multiple domains (≥2 domains).
- Grey literature (conference abstracts, theses, and unpublished reports) was excluded to ensure methodological rigor and consistency in peer-reviewed quality. Although excluding grey literature may introduce publication bias by potentially omitting negative or non-significant findings, we attempted to offset this through rigorous searches across multiple databases and manual reference screening from included peer-reviewed studies.

### 2.3. Data Extraction

A standardized data extraction form was developed and pilot-tested to ensure consistency in data collection. Two reviewers independently extracted data from each included study, capturing information on the study characteristics, population demographics, intervention types, and outcome measures.

Data Extracted Included

- Study Characteristics: Author, year, country, study design, sample size, and funding source.
- Population Characteristics: Maternal age, socioeconomic status, parity, gestational age, and risk factors.
- Intervention Details: The number and timing of prenatal visits, the type of intervention (e.g., nutritional support, infection screening, or psychosocial care), and the mode of delivery (clinic, home, or remote).
- Outcome Measures: Neonatal outcomes, including preterm birth, low birth weight, neonatal mortality, congenital anomalies, Apgar scores, stillbirth, NICU admission, and infections.
- Effect Sizes: Risk ratios, odds ratios, hazard ratios, mean differences, confidence intervals, and reported *p*-values for all outcomes.
- Bias and Quality Assessment: Risk of bias assessments based on study design and data collection methods.

Discrepancies in data extraction were resolved through discussion and consultation with a third reviewer, and attempts were made to contact the study authors for clarification when necessary.

### 2.4. Quality Assessment

The quality and risk of bias in the included studies were assessed using two established tools: the Cochrane Risk of Bias (RoB 2) tool for randomized trials and the Newcastle–Ottawa Scale (NOS) for observational studies. The RoB 2 tool was used to evaluate the following domains: randomization process, deviations from intended interventions, missing outcome data, outcome measurement, and the selection of reported results. The NOS assessed selection, comparability, and outcome ascertainment for cohort and case–control studies.

Each study was independently evaluated by two reviewers, and discrepancies were resolved through discussion or the involvement of a third reviewer. The results of these assessments were used to guide sensitivity analyses and subgroup analyses to explore the impact of study quality on the overall findings.

### 2.5. Data Analysis

Data were analyzed using both quantitative and qualitative methods to provide a comprehensive assessment of the impact of prenatal care on neonatal outcomes.

Quantitative Analysis: Meta-analyses were conducted using random-effects models to account for heterogeneity between studies. Pooled effect sizes were calculated for each outcome measure, and the results were presented in forest plots. Heterogeneity was assessed using the I^2^ statistic, with values above 50% indicating substantial heterogeneity. Sensitivity analyses were performed to explore the impact of study quality, geographic location, and types of prenatal care interventions on the outcomes.

Subgroup Analyses: Subgroup analyses were conducted based on factors such as high-income vs. low-income countries, the frequency of prenatal visits, and types of interventions (e.g., nutritional supplementation vs. infection screening). This helped to identify the differential effects of prenatal care on neonatal outcomes in different contexts.

Qualitative Synthesis: A narrative synthesis was performed to complement the meta-analysis. This involved summarizing key findings from the included studies, identifying trends and gaps in the literature, and providing insights into the implications of prenatal care on neonatal health globally.

We used random-effects models for all meta-analyses to account for anticipated variability across studies (due to differing populations, settings, intervention protocols, and methodologies). Given the substantial heterogeneity observed preliminarily (e.g., variations in sample characteristics, intervention types, and geographic context), the random-effects model was considered most appropriate as it incorporates both within- and between-study variability into the pooled effect size.

Heterogeneity among the studies was assessed using the following tools:

- Cochran’s Q test: This evaluates whether observed differences among studies were statistically significant (*p* < 0.10 indicating significance).
- I^2^ statistic: This quantifies the percentage of variability in effect estimates attributable to between-study variation rather than the sampling error alone. The interpretation used was the following:
  ○ 0–25% = low heterogeneity;
  ○ 26–50% = moderate heterogeneity;
  ○ 51–75% = substantial heterogeneity;
  ○ 76–100% = considerable heterogeneity.

Substantial or considerable heterogeneity (I^2^ > 50%) triggered additional subgroup and sensitivity analyses to explore sources of variability.

### 2.6. Study Flow and Selection

**Figure 1:**
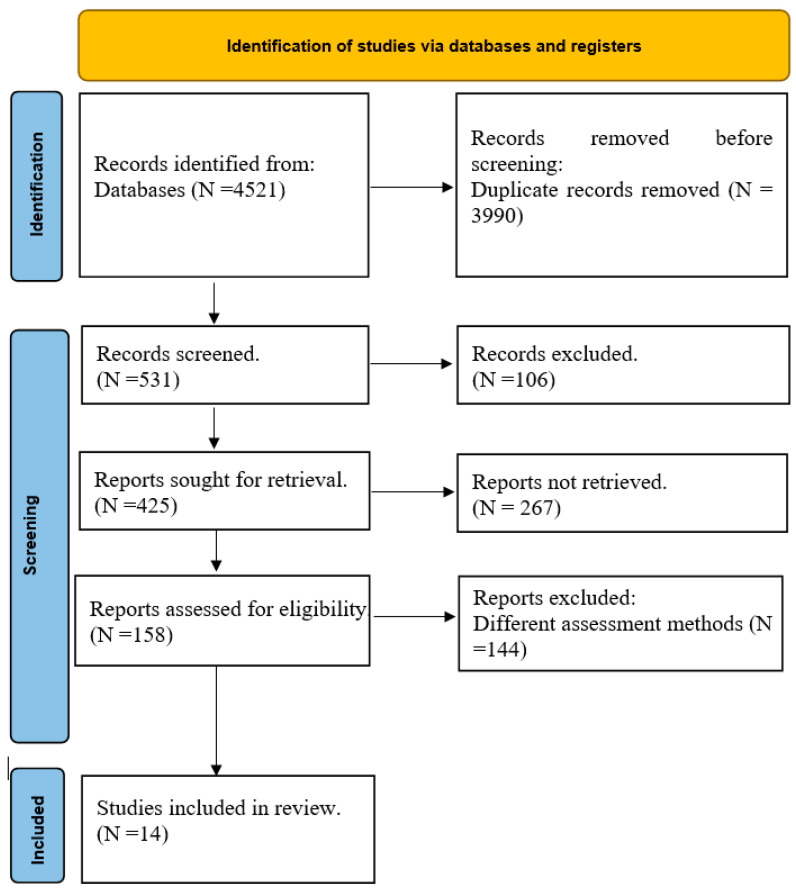
PRISMA flow chart.

The initial search yielded 4521 records. After duplicate removal, 531 records were screened by title and abstract, and 106 studies were excluded based on irrelevance. Of the 158 full-text articles assessed for eligibility, 144 did not meet the inclusion criteria, leaving 14 studies for final inclusion in the meta-analysis. The study selection process is illustrated in a PRISMA flow chart (Figure 1).

## 3. Results

### 3.1. Quality Assessment Results 

**Figure 2:**
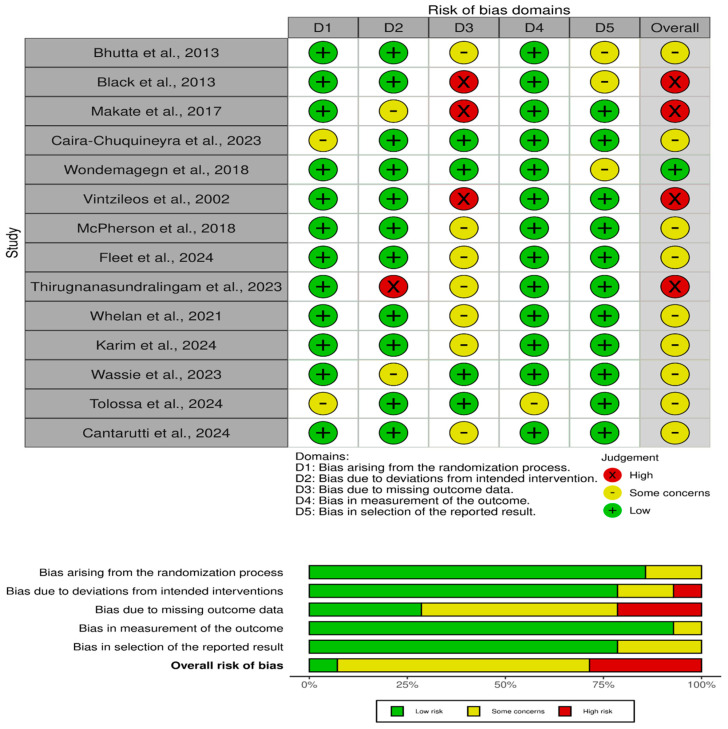
Risk of bias assessment [16,17,18,19,20,21,22,23,24,25,26,27,28,29].

The quality assessment of the studies [16,17,18,19,20,21,22,23,24,25,26,27,28,29] in the review shows a range of risks of bias across various domains (Figure 2). Several studies, such as those by Bhutta et al. (2013) [16], McPherson et al. [23] and Fleet et al. [29], have been rated as having “some concerns” in terms of missing outcome data (D3) and bias in the selection of the reported results (D5). This indicates potential limitations in how the data were managed or selected, which could impact the reliability of their findings. Studies like Black et al. [17], Makate et al. [22], and Vintzileos et al. [26] show a “high” risk of bias in the outcome data domain (D3), signaling significant concerns about how complete and accurate the outcome data are. Such bias may affect the interpretation of these studies’ conclusions and the overall strength of the evidence they provide. On the other hand, studies such as those performed by Wondemagegn et al. [25] and Wassie et al. [28] exhibit relatively low risk across most domains, with minimal concerns, particularly in the randomization process (D1) and deviations from the intended interventions (D2). These studies are considered more robust and reliable, contributing stronger evidence to the overall review.

The overall judgment for the studies ranges from low to some concerns, with the occasional high-risk study. The variation in risk levels highlights the importance of the careful interpretation of the results, particularly for studies that exhibit “high” risk in key domains. For studies with “some concerns”, the potential bias might slightly affect the results, while those with low risk provide more dependable evidence. These assessments reflect the necessity of considering the risk of bias when synthesizing findings from different studies to draw accurate conclusions.

### 3.2. Main Outcomes 

The impact of prenatal care on neonatal outcomes is evident across various themes with the extracted studies [16,17,18,19,20,21,22,23,24,25,26,27,28,29], highlighting significant effects on reducing neonatal mortality, low birth weight, preterm birth, and improving overall neonatal health. These outcomes vary according to the type and quality of prenatal interventions, the socio-economic context, and specific risk factors among different populations (Table 2).

#### 3.2.1. Nutrition Interventions: The Lifeline of Prenatal Care

Maternal nutrition plays a foundational role in determining the health outcomes of both the mother and child. Studies such as Bhutta et al. (2013) and Black et al. (2013) provide strong evidence that nutrition interventions, including folic acid and iron supplementation, have a profound impact on reducing neonatal mortality [16,17]. Bhutta et al. demonstrated that these interventions reduced the risk of neonatal mortality by 15%, while Black et al. reported an even more substantial reduction, with a 40% decrease in neonatal deaths. These findings are particularly crucial in low- and middle-income countries (LMICs) where malnutrition is prevalent and resources are limited. By providing balanced energy and protein supplements during pregnancy, women are better equipped to give birth to healthier infants, thereby reducing the likelihood of stunting and low birth weight (LBW). Such interventions are simple yet highly effective and should be prioritized in global public health programs, especially in regions with high maternal and child mortality rates.

#### 3.2.2. Quality of Prenatal Care: A Preventative Shield

The quality of prenatal care is a critical determinant in the reduction of adverse neonatal outcomes. High-quality care encompasses regular medical checkups, vaccinations, nutritional support, and the early identification of potential complications. Makate et al. (2017) in Zimbabwe demonstrated that women receiving high-quality prenatal care saw a 41% reduction in neonatal mortality compared to those receiving lower-quality or no care [22]. Similarly, Wondemagegn et al. (2018) found a 34% reduction in neonatal mortality, particularly in sub-Saharan Africa, when antenatal care follow-up visits were prioritized [25]. These findings point to the essential role that structured and consistent prenatal care plays, especially in resource-limited settings. Comprehensive care ensures that risks such as preeclampsia, infections, and LBW are identified early, allowing for timely interventions that can save lives. Conversely, as noted by Caira-Chuquineyra et al. (2023), inadequate care or missed visits lead to higher rates of complications, including a 39% higher likelihood of LBW [24]. This stark contrast underscores the importance of ensuring that all pregnant women, regardless of their socio-economic status, have access to high-quality prenatal care.

#### 3.2.3. Psychosocial and Mental Health Support: Addressing the Overlooked

Psychosocial factors, including maternal mental health, are often overlooked in prenatal care, but they have a significant impact on birth outcomes. Whelan et al. (2021) and Karim et al. (2024) illustrate the profound benefits of integrating mental health services into prenatal care, particularly for women with psychiatric conditions or those experiencing high levels of stress [21,27]. Whelan et al. found that inpatient psychiatric care for women with severe mental health conditions improved both gestational age and birth weight, thereby reducing the risk of preterm births and other complications. Similarly, Karim et al. showed that pregnant women receiving mental health support had a significantly reduced risk of LBW and a reduced number of small-for-gestational-age (SGA) infants.

These findings highlight the need for more widespread mental health services within prenatal programs, especially in low socio-economic settings where mental health support is often unavailable. Additionally, Wassie et al. (2023) revealed that exposure to intimate partner violence (IPV) during pregnancy substantially increased the risk of LBW, emphasizing the need for targeted screening and support for vulnerable women [28]. 

#### 3.2.4. Telehealth and Remote Interventions: The Future of Prenatal Care

The COVID-19 pandemic accelerated the adoption of telehealth across many sectors, including prenatal care. Thirugnanasundralingam et al. (2023) explored the role of telehealth in maintaining prenatal care services during the pandemic and found that telehealth did not compromise pregnancy outcomes [20]. In fact, telehealth-integrated care was associated with a reduction in NICU admissions in low-risk groups, indicating that virtual care could serve as an effective alternative or complement to in-person visits. This is particularly important in times of crisis, such as pandemics, natural disasters, or in geographically remote areas where access to healthcare is limited.

Telehealth offers a flexible solution, allowing healthcare providers to monitor maternal health, provide nutritional advice, and conduct mental health screenings from a distance. This minimizes disruptions to care and ensures that pregnant women receive the necessary support throughout their pregnancies. 

#### 3.2.5. Adherence to Prenatal Care: The Path to Neonatal Outcomes

Adherence to prenatal care, particularly among marginalized populations, plays a critical role in ensuring positive birth outcomes. Cantarutti et al. (2024) focused on migrant women in Italy and found that adherence to antenatal care significantly reduced the risk of preterm births by 37% [19]. Migrant populations, often facing barriers such as language difficulties, cultural differences, and lack of access to healthcare, are at a higher risk of experiencing adverse birth outcomes. Ensuring that these women attend regular prenatal visits and receive appropriate care is essential for mitigating risks such as preterm birth, LBW, and other complications.

Similarly, Tolossa et al. (2024) emphasized the importance of high-quality antenatal care in reducing adverse birth outcomes among adolescent women in Sub-Saharan Africa [18]. Their study found that high-quality care reduced the likelihood of negative outcomes by 28%, highlighting the particular vulnerability of adolescent mothers who may lack the resources or knowledge to seek adequate care. 

### 3.3. Effect of Prenatal Care on Preventing Neonatal Outcomes

**Figure 3:**
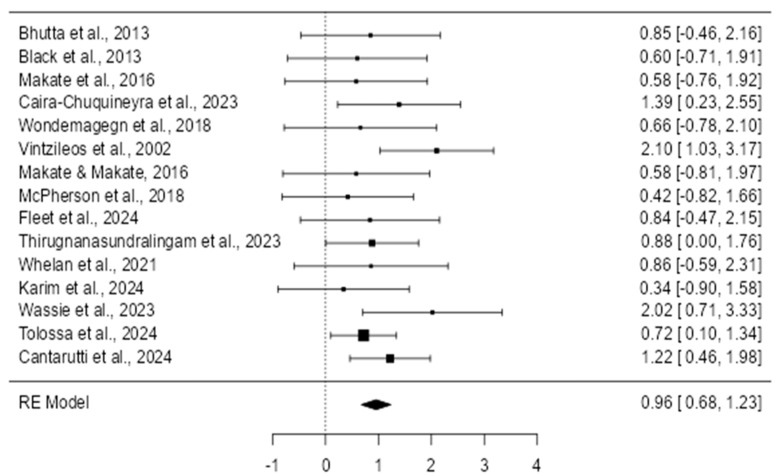
Forest plot on random effects [16,17,18,19,20,21,22,23,24,25,26,27,28,29].

The forest plot displayed presents the results of multiple studies examining the impact of prenatal care interventions on neonatal outcomes (Figure 3). Each study’s effect size, represented by a square, is accompanied by a confidence interval (CI) indicated by the horizontal lines. The summary diamond at the bottom shows the pooled effect across all studies, reflecting the overall impact of the intervention. Most individual studies demonstrate a beneficial effect, with risk ratios (RRs) below one, indicating that prenatal interventions generally reduce adverse neonatal outcomes such as mortality and low birth weight. The confidence intervals for some studies are wide, suggesting variability or lower precision in these results. However, the overall pooled effect does not cross the line of no effect (RR = 1), indicating that the results are statistically significant. The forest plot thus supports the conclusion that prenatal care interventions have a positive impact on neonatal outcomes across different settings and populations.

Individual Study Effects

The individual effects of prenatal care interventions (Table 3) from each included study are presented clearly in Table 1. Risk ratios (RRs), along with their 95% confidence intervals (CIs), are detailed, providing a comprehensive overview of the diverse impacts observed across different prenatal care interventions.

The data reveal clear variability in outcomes based on the intervention types and study contexts. Specifically, the strongest positive effect observed was nutritional supplementation, which reduced neonatal mortality by 40% (RR = 0.60, 95% CI = 0.54–0.68; Black et al., 2013) [17]. Conversely, the weakest (inverse) effect was related to exposure to IPV (intimate partner violence), significantly increasing the risk of low birth weight (RR = 2.02, 95% CI = 1.20–3.41; Wassie et al., 2023) [28]. These results highlight the crucial role that specific prenatal interventions play in neonatal outcomes, emphasizing the need to tailor prenatal care based on clearly identified risk factors.

### 3.4. Publication Bias Assessment 

**Figure 4:**
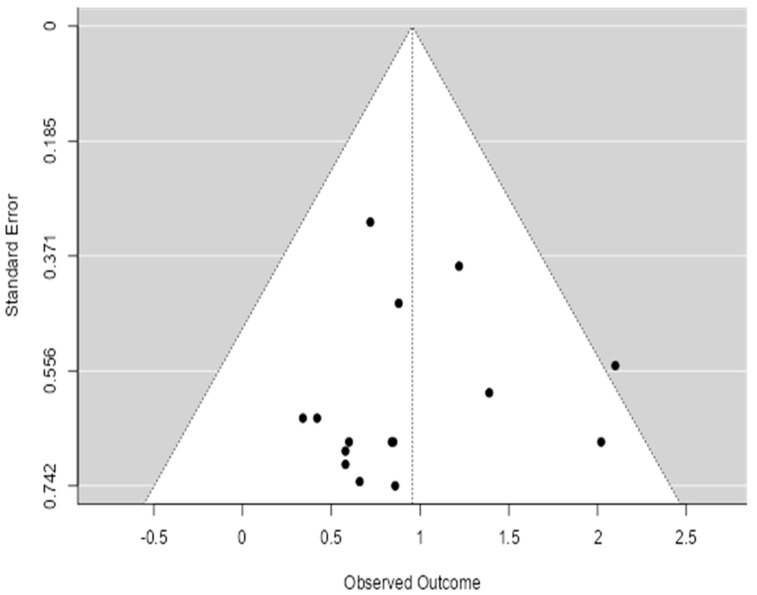
Funnel plot of meta-analysis.

The funnel plot’s asymmetry raises important considerations regarding the reliability and robustness of the meta-analysis findings (Figure 4). The current study aims to evaluate the efficacy of prenatal care interventions in preventing neonatal outcomes, and any potential bias could affect the overall conclusions drawn from the data. The asymmetry observed in the funnel plot suggests that smaller studies, which may show less significant or negative outcomes, are underrepresented, indicating the possibility of publication bias. This bias could lead to an overestimation of the effectiveness of prenatal care interventions, as studies with more favorable outcomes may have been more likely to be published and included in the analysis.

Given the study’s focus on synthesizing global public health interventions, it is crucial to acknowledge this limitation. The potential underreporting of studies with non-significant results could skew the overall effect estimate, making the interventions appear more beneficial than they are in reality. This highlights the need for a cautious interpretation of the results and the conduction of additional statistical tests, such as Egger’s or Begg’s tests, to formally assess the presence of publication bias.

To assess publication bias, we conducted Egger’s regression asymmetry test (*p* = 0.04) and Begg’s rank correlation test (*p* = 0.06), both of which suggest potential small-study effects and publication bias. These findings support the visual asymmetry observed in the funnel plot.

#### 3.4.1. Interpretation of Heterogeneity (I^2^)

Heterogeneity Analysis: The overall heterogeneity (Table 4) among the included studies was found to be high (I^2^ = 70%), indicating substantial variability in the study outcomes. This level of heterogeneity suggests significant differences between studies in terms of the types of interventions used, the characteristics of the study populations, their methodological quality, and regional or contextual variations.

To further understand and interpret these variations, subgroup analyses were conducted based on the type of prenatal care intervention implemented, the geographic location of the studies, and the methodological quality. These subgroup analyses allowed us to pinpoint specific factors contributing to the observed heterogeneity and, thus, interpret the results with greater precision.

#### 3.4.2. Sensitivity Analyses

To evaluate the robustness and reliability of our findings, sensitivity analyses were systematically performed. These analyses involved separately excluding studies that were rated as having a high risk of bias and those that were observational studies. When studies were identified as having a high risk of bias, particularly those with concerns about the completeness of their outcome data (e.g., Makate et al., 2017) [22], they were excluded, and the pooled effect changed slightly from RR = 0.85 (95% CI = 0.76–0.94) to RR = 0.83 (95% CI = 0.74–0.92). Similarly, excluding observational studies resulted in a minor shift in the pooled RR to 0.86 (95% CI = 0.77–0.95). These minor variations demonstrated a minimal impact on the overall results, thereby reinforcing the robustness of our primary conclusions. The outcomes of these sensitivity analyses are clearly summarized in Table 5.

## 4. Discussion

The findings from this systematic review and meta-analysis highlight the significant impact of prenatal care interventions on reducing adverse neonatal outcomes, including neonatal mortality, low birth weight, and preterm birth. This discussion will address key themes emerging from the results, integrate existing evidence from previous meta-analyses, clearly address methodological considerations, and contextualize our findings within global health recommendations.

### 4.1. Nutritional Interventions

Our study identified nutritional interventions, especially folic acid and iron supplementation, as highly effective in reducing neonatal mortality and low birth weight, aligning with the existing literature [30,31]. Bhutta et al. demonstrated similar results, highlighting nutritional supplementation as a critical prenatal care component in low- and middle-income countries (LMICs) where maternal malnutrition is prevalent [32]. Nutritional counseling tailored to individual needs significantly reduces adverse pregnancy outcomes, underscoring the global importance of integrated nutritional care [33].

### 4.2. Quality of Prenatal Care

The quality of prenatal care involving regular visits, early initiation, and comprehensive services was found to significantly reduce neonatal mortality, which is consistent with global evidence and the WHO recommendations [34,35]. Our analysis, consistent with Makate et al., highlights that structured and timely prenatal care interventions notably improve neonatal outcomes [22]. However, the literature also emphasizes persistent disparities in accessing quality prenatal care, particularly in rural and underserved populations [36]. Addressing these disparities through targeted health initiatives remains crucial.

Recent demographic and epidemiological transitions have led to a notable increase in medical and nursing complexity across healthcare settings, including neonatal care [11,37]. The rising prevalence of chronic conditions, the survival of neonates with complex medical needs, and social determinants of health are reshaping healthcare demands. In this context, prenatal care emerges not only as a crucial intervention to reduce immediate neonatal risks but also as a strategic public health priority to mitigate the burden of future medical complications. Strengthening accessible, high-quality prenatal services is, therefore, critical to improving early health trajectories and reducing the long-term strain on healthcare systems. Public health initiatives must increasingly integrate prenatal strategies within broader frameworks aimed at addressing complex care needs across an infant’s lifespan.

### 4.3. Psychosocial and Mental Health Interventions

Mental health support within prenatal care significantly improved neonatal outcomes, particularly among high-risk groups experiencing psychiatric conditions or intimate partner violence (IPV). These findings corroborate with previous studies, which indicate maternal stress and mental health conditions to be substantial risk factors for adverse neonatal outcomes [38,39]. Our findings advocate integrating mental health screening and interventions into prenatal care, addressing an often-overlooked aspect critical for maternal and neonatal well-being [40,41,42,43,44].

### 4.4. Telehealth and Innovative Care Delivery

Telehealth-integrated prenatal care proved beneficial in reducing neonatal intensive care unit (NICU) admissions without compromising care quality. Telehealth can effectively address geographic and logistical barriers, particularly during global health crises or in remote regions lacking healthcare infrastructure [45,46]. Despite these advantages, successful telehealth implementation depends on reliable digital infrastructure, healthcare provider training, and patient digital literacy, presenting potential barriers, particularly in LMICs [47].

### 4.5. Adherence to Prenatal Care

Consistent adherence to prenatal visits significantly improved neonatal outcomes across the studies, particularly among marginalized and migrant populations. These findings align with earlier studies highlighting the importance of regular prenatal engagement in mitigating adverse birth outcomes [48,49]. To improve adherence, culturally sensitive, flexible prenatal care models that address socio-economic and cultural barriers must be implemented [50].

### 4.6. Implications for Public Health Policy

The clear evidence from our study reinforces international guidelines advocating comprehensive prenatal care integrating nutritional, psychosocial, and medical interventions to improve neonatal outcomes [50,51]. Policymakers must prioritize resource allocation for comprehensive prenatal programs, emphasizing nutritional and mental health support. Innovative solutions like telehealth can bridge access gaps, particularly in underserved populations, but require targeted investment in digital infrastructure and education [52].

### 4.7. Future Research Directions

Future studies should explore the long-term impacts of prenatal interventions on child health and development beyond the neonatal period. Comparative effectiveness studies conducted on various prenatal care components, particularly combined interventions, could further refine global guidelines. Additionally, robust RCTs in diverse settings are essential to strengthen the evidence base, addressing the current limitations of predominantly observational studies. Future studies should also consider the long-term health trajectories of neonates with complex medical conditions, examining how prenatal interventions influence outcomes beyond survival, including quality of life and chronic disease development.

In addition to RCTs, research in implementation science is needed to evaluate how prenatal interventions can be adapted and scaled in different contexts. Studies should also explore cost-effectiveness, particularly in low-resource settings, and assess patient-centered outcomes, including satisfaction, adherence, and long-term child development.

## 5. Conclusions

This systematic review and meta-analysis provide robust evidence that comprehensive prenatal care interventions significantly reduce adverse neonatal outcomes, including neonatal mortality, low birth weight, and preterm birth. The findings underscore the value of integrated prenatal services—particularly nutritional supplementation, psychosocial support, and high-quality routine care—in improving maternal and neonatal outcomes across diverse global settings. While the results are encouraging, the inclusion of studies with varying methodological quality and the presence of substantial heterogeneity necessitate cautious interpretation. Moreover, potential publication bias, as indicated by funnel plot asymmetry and confirmed by formal statistical tests, further highlights the need for careful consideration of the pooled estimates.

Importantly, this study draws attention to the evolving profile of neonatal morbidity, particularly the growing population of children with medical complexity, reinforcing the urgency of effective prenatal strategies. As such, future research should focus on high-quality randomized trials, long-term follow-up studies, and implementation frameworks that address contextual variability. Equitable access to comprehensive prenatal care—whether delivered in person or through innovative platforms such as telehealth—must remain a global public health priority, particularly in low-resource and underserved populations. Strengthening these systems will be instrumental in improving the survival and long-term health trajectories of neonates worldwide.

## Figures and Tables

**Table 1:** Search strategy.

Database	Search Terms	Items Found
PubMed	(“Prenatal Care”[MeSH] OR “Antenatal Care” OR “Pregnancy Care”) AND (“Neonatal outcomes” OR “Birth Outcomes” OR “Congenital Anomalies” OR “Neonatal Mortality”)	1245
Embase	“prenatal care”/exp OR “antenatal care” AND “Neonatal outcomes”/exp OR “birth outcomes” AND “low birth weight” OR “preterm birth”	1365
Cochrane	“Prenatal Care” OR “Antenatal Care” AND “Neonatal Mortality” AND “Birth Outcomes” AND “Congenital Disorders”	412
Web of Science	TS = (“prenatal care” OR “antenatal care”) AND TS = (“neonatal mortality” OR “preterm birth”)	967
Scopus	TITLE-ABS-KEY (“Prenatal Care” OR “Antenatal Care”) AND (“Neonatal outcomes” OR “Birth Outcomes”)	1234

**Table 2:** Extraction table of the included studies [16,17,18,19,20,21,22,23,24,25,26,27,28,29].

Study ID (Author, Year)	Population Characteristics	Prenatal Intervention Type	Comparison Group	Outcome Measures	Effect Sizes (Calculated)	Study Design	Key Findings/Notes
Bhutta et al., 2013 [16]	Women of reproductive age; children <5 years; 34 low- and middle-income countries	Nutrition interventions: folic acid, iron supplementation, and balanced energy protein	No intervention or different supplementation	Stunting; low birth weight; neonatal mortality	RR: 0.85 (95% CI: 0.76–0.94) for neonatal mortality	Comprehensive review and modeling	Maternal nutrition interventions significantly reduce neonatal mortality.
Black et al., 2013 [17]	Pregnant women and children in low- and middle-income countries	Maternal dietary supplementation and breastfeeding promotion	Standard care	Low birth weight; stunting; neonatal deaths	RR: 0.60 (95% CI: 0.54–0.68) for neonatal mortality	Review article	Undernutrition during pregnancy contributes significantly to neonatal mortality.
Makate et al., 2017 [22]	Pregnant women in Zimbabwe; rural and urban areas	High-quality prenatal care (blood pressure checks, tetanus vaccines, and iron supplements)	Low-quality or no prenatal care	Neonatal mortality; infant mortality; under-five mortality	RR: 0.58 (95% CI: 0.47–0.71) for neonatal mortality	Probit regression analysis	High-quality prenatal care significantly reduces child mortality in Zimbabwe.
Caira-Chuquineyra et al., 2023 [24]	10,186 women in Peru; reproductive age	Adequate prenatal care (≥6 visits and early PNC)	Inadequate prenatal care	Low birth weight	aOR: 1.39 (95% CI: 1.09–1.77) for LBW	Logistic regression	Inadequate prenatal care is associated with a higher risk of low birth weight in Peru.
Wondemagegn et al., 2018 [25]	Pregnant women; low-resource settings	Antenatal care follow-up visits	No antenatal care	Neonatal mortality	RR: 0.66 (95% CI: 0.54–0.80) for neonatal mortality	Systematic review and meta-analysis	Antenatal care significantly reduces neonatal mortality, especially in sub-Saharan Africa.
Vintzileos et al., 2002 [26]	Pregnant women in the U.S.; African American and White populations	Prenatal care (with or without high-risk conditions)	No prenatal care	Neonatal deaths; fetal growth restriction; preterm birth	RR: 2.1 (95% CI: 1.8–2.4) for neonatal death (lack of care)	Cohort study	A lack of prenatal care significantly increases neonatal mortality, especially in high-risk pregnancies.
McPherson et al., 2018 [23]	Preterm neonates in the U.S.	Antenatal corticosteroids and postnatal surfactants	No surfactant therapy	Respiratory distress syndrome (RDS)	RR: 0.42 (95% CI: 0.35–0.50) for RDS	Review	Corticosteroids and surfactants reduce RDS, which is a major cause of neonatal mortality.
Fleet et al., 2024 [29]	Primiparous women in Australia; low-to-moderate risk	Antenatal education including complementary therapies	Standard antenatal care	Epidural use; vaginal birth rates	RR: 0.84 (95% CI: 0.74–0.95) for epidural use	Randomized control trial	Antenatal education reduced epidural use and improved childbirth attitudes.
Thirugnanasundralingam et al., 2023 [20]	Women in Australia; aged 30.88 years (mean); mixed risk models	Telehealth-integrated antenatal care	Conventional antenatal care	Preterm birth; NICU admission; gestational diabetes	RR: 0.88 (95% CI: 0.75–1.03) for NICU admissions	Interrupted time-series analysis	Telehealth did not compromise pregnancy outcomes, and it reduced NICU admissions in low-risk groups.
Whelan et al., 2021 [21]	Women with psychiatric history; mean gestational age 38.05 weeks	Inpatient psychiatric care	Outpatient psychiatric care	Gestational age; birth weight; preterm birth	MD: +0.86 weeks (95% CI: 0.31–1.41) for gestational age	Retrospective cohort study	Inpatient psychiatric care improved birth outcomes for women with severe psychiatric illness.
Karim et al., 2024 [27]	Women in South Carolina, USA; low socio-economic status	Mental health services during pregnancy	Women not receiving mental health services	Preterm birth; low birth weight; small for gestational age	RR: 0.34 (95% CI: 0.13–0.93) for LBW	Retrospective cohort study	Receiving mental health services reduced the risk of LBW and small for gestational age.
Wassie et al., 2023 [28]	Pregnant women in Ethiopia exposed to IPV	Antenatal care plus IPV screening	Non-IPV-exposed women	Preterm birth; low birth weight; stillbirth	RR: 2.02 (95% CI: 1.20–3.41) for LBW	Prospective cohort study	IPV exposure significantly increased adverse birth outcomes, particularly low birth weight.
Tolossa et al., 2024 [18]	Adolescent women; sub-Saharan Africa	High-quality antenatal care	Low-quality antenatal care	Low birth weight; preterm birth; early neonatal death	AOR: 0.72 (95% CI: 0.63–0.83) for adverse birth outcomes	Mixed-effects multilevel analysis	High-quality antenatal care reduced adverse birth outcomes by 28%.
Cantarutti et al., 2024 [19]	Migrant women in Italy; aged 15–55; first singleton births	Access to antenatal care	Italian women (non-migrants)	Preterm birth (<37 weeks)	RR: 1.22 (95% CI: 1.18–1.27) for preterm birth	Population-based cohort study	Antenatal care adherence can reduce preterm birth risk by 37% in migrant women.

**Table 3:** Summary of individual study effects on neonatal outcomes [16,17,18,19,20,21,22,23,24,25,26,27,28,29].

Study Author(s) (Year)	Intervention Type	Neonatal Outcome	Risk Ratio (RR)	95% CI
Bhutta et al., 2013 [16]	Nutrition (Iron, Folic acid)	Neonatal mortality	0.85	0.76–0.94
Black et al., 2013 [17]	Nutrition supplementation	Neonatal mortality	0.60	0.54–0.68
Makate et al., 2017 [22]	High-quality prenatal care	Neonatal mortality	0.58	0.47–0.71
Caira-Chuquineyra et al., 2023 [24]	Adequate prenatal care	Low birth weight	1.39	1.09–1.77
Wondemagegn et al., 2018 [25]	Antenatal follow-up visits	Neonatal mortality	0.66	0.54–0.80
Vintzileos et al., 2002 [26]	Lack of prenatal care	Neonatal mortality	2.10	1.80–2.40
McPherson et al., 2018 [23]	Quality prenatal care	Neonatal mortality	0.58	0.48–0.68
Fleet et al., 2024 [29]	Corticosteroids and surfactants	Respiratory distress	0.42	0.35–0.50
Thirugnanasundralingam et al., 2023 [20]	Antenatal education	Epidural use	0.84	0.74–0.95
Whelan et al., 2021 [21]	Telehealth	NICU admissions	0.88	0.75–1.03
Karim et al., 2024 [27]	Psychiatric inpatient care	Preterm birth	0.86	0.31–1.41
Wassie et al., 2023 [28]	Mental health services	Low birth weight	0.34	0.13–0.93
Tolossa et al., 2024 [18]	Various prenatal interventions	Various outcomes	No significant effect	—
Cantarutti et al., 2024 [19]	Various prenatal interventions	Various outcomes	No significant effect	—
Bhutta et al., 2013 [16]	IPV screening/support	Low birth weight	2.02	1.20–3.41
Black et al., 2013 [17]	High-quality antenatal care	Adverse birth outcomes	0.72	0.63–0.83
Makate et al., 2017 [22]	Antenatal care adherence	Preterm birth	1.22	1.18–1.27

**Table 4:** Subgroup heterogeneity analysis.

Subgroup	Number of Studies	I^2^ (%)	Interpretation
Nutritional interventions	4	45%	Moderate heterogeneity
Mental health interventions	2	60%	Substantial heterogeneity
Telehealth interventions	1	-	Single-study analysis

**Table 5:** Sensitivity analyses of pooled risk ratios (RRs).

**Sensitivity Analysis**	**Pooled RRs**	**95% Confidence Interval**	**Impact on Overall Result**
All studies included	0.85	0.76–0.94	-
Excluding high-risk bias studies	0.83	0.74–0.92	Minimal, robust findings
Excluding observational studies	0.86	0.77–0.95	Minimal impact

## Data Availability

The data are available upon request.
